# General practitioners’ perspectives on chronic care consultations for patients with a history of cancer: a qualitative interview study

**DOI:** 10.1186/s12875-019-1009-5

**Published:** 2019-08-27

**Authors:** Anne Beiter Arreskov, Anette Hauskov Graungaard, Mads Toft Kristensen, Jens Søndergaard, Annette Sofie Davidsen

**Affiliations:** 1 Department of Public Health, University of Copenhagen, Copenhagen, Denmark; 2 Institute of Public Health, University of Southern Denmark, Odense, Denmark; 3 The Research Unit for General Practice and Section of General Practice, Department of Public Health, University of Copenhagen, Øster Farimagsgade 5, P.O. Box 2099, DK-1014 Copenhagen, Denmark

**Keywords:** Primary care, General practitioners, Prioritizing, Agenda, Chronic care, Cancer, Normalcy

## Abstract

**Background:**

General practitioners (GPs) are responsible for managing chronic care in the growing population of patients with comorbid chronic conditions and cancer. Studies have shown, however, that cancer patients are less likely to receive appropriate chronic care compared to patients without cancer. Patients say that how GPs engage in the care of comorbidities influences their own priority of these conditions. No studies have explored GPs’ attitudes to and prioritization of chronic care in patients who have completed primary cancer treatment. This study aims to explore GPs’ experiences, prioritization of, and perspectives on treatment and follow-up of patients with cancer and comorbidity.

**Methods:**

Semi-structured interviews were conducted during 2016 with 13 GPs in Region Zealand in Denmark. We used Systematic Text Condensation in the analysis.

**Results:**

All participating GPs said that chronic care in patients with a history of cancer was a high priority, and due to a clear structure in their practice, they experienced that few patients were lost to follow-up. Two different approaches to chronic care consultations were identified: one group of GPs described them as imitating outpatient clinics, where the GP sets the agenda and focuses on the chronic condition. The other group described an approach that was more attuned to the patient’s agenda, which could mean that chronic care consultations served as an “alibi” for the patients to disclose other matters of concern.

Both groups of GPs said that chronic care consultations for these patients supported *normalcy*, but in different ways. Some GPs said that offering future appointments in the chronic care process gave patients hope and a sense of normalcy. Other GPs strove for normalcy by focusing exclusively on the chronic condition and dealing with cancer as cured.

**Conclusions:**

The participating GPs gave a high priority to chronic care in patients with a history of cancer. Some GPs, however, followed a rigorous agenda. GPs should be aware that a very focused and biomedical approach to chronic care might increase fragmentation of care and collide with a holistic and patient-centered approach. It could also affect GPs’ self-perception of their role and the core values of general practice.

## Background

In Denmark, general practitioners (GPs) are responsible for providing care for patients with chronic conditions including follow-up on certain types of cancer [1, 2]. Advancing age is associated with an increased prevalence of cancer and comorbidity comprising chronic conditions such as diabetes (DM), cardiovascular disease (CVD) and chronic obstructive pulmonary disease (COPD).

Patients with cancer and comorbidity have increased all-cause mortality compared to cancer patients without comorbidities [3–7]. Further, they are confronted with various challenges, for instance, the organization of care when treatment is shared between general practice and hospitals [8–10]. This may hamper the coherence of care [11, 12] and continuity in the patient’s contact with their GP [13]. A recent Scottish study showed that patients with more than one chronic condition were more likely to miss their general practice appointments [14]. Other studies from the US and the UK have shown that patients with cancer and chronic conditions were less likely to receive preventive services and appropriate monitoring of e.g. DM, COPD and CVD compared to patients without cancer [15–19].

One explanation could be that both patients and health care providers give the comorbidities a lower priority than the cancer treatment [5, 20–24]. In a study of patients with comorbidity and a history of cancer, however, patients reported that cancer did not change their attendance at chronic care consultations in the long run. Further, chronic care consultations were described as a well-liked, predictable, everyday routine, and the patients appreciated the relationship with their GP as well as the staff in the practice [25]. This underlines some of the core values of general practice, which defines itself in terms of relationships, routines and continuity developed over time [26]. The core values of general practice have been described as knowing the patient as a person, and although patients may have diseases in common, they are unique in their responses to disease [27, 28]. Enid Balint described this understanding of the patient as a unique human being as “patient-centered” [29]; and Byrne and Long [30] described a special general practice consultation style where the GP used the patient’s knowledge and point of view to guide the interaction. McWhinney described this approach as an endeavor to enter the patient’s world and *“see the illness through the patient’s eyes”* [31] to understand the meaning of the illness for the patient [32].

General practice, however, has changed over the years [33, 34] and a growing set of demands is placed on primary care, including clinical guidelines that describe expectations to the content of certain consultations, e.g., chronic care management [35]. This might result in a shift towards a more biomedical, single-disease-oriented model of care, where patients are increasingly identified by disease labels and their care is determined by those labels [36]. The consequences are a challenge to the patient-centered role of GPs, who have reported a risk of giving the patient’s other problems a lower priority when the consultations were too “guideline-driven” [37, 38]. A focus on biomedical aspects might, therefore, hinder creating a space for exploring the patient’s needs [38, 39].

Moreover, due to, e.g., a fear of wasting the GP’s time [40], disclosure of emotional problems can be difficult for patients. Joensson et al. reported that older patients with multiple chronic conditions refrained from the disclosure of problems to avoid putting themselves in a position perceived to be “*inferior”* [41]. Pollock et al. [42] reported that some patients jeopardized their chances of receiving attention for their distress by keeping up a façade [43]. GPs, therefore, might need to have a more proactive approach to make patients articulate their real agenda [44].

In the study about priorities in patients with a history of cancer and comorbidities, patients said that it was not the specific disease that caused concern, but symptoms that impaired their function. These patients highly valued their GP’s acquaintance with them as people, and the GP’s engagement in the care of their comorbidities influenced how they prioritized their chronic conditions [25].

No studies, however, have explored GPs’ approaches to and prioritization of chronic care in patients with a history of cancer, and how the cancer disease might affect chronic care consultations. This study, therefore, aimed to explore GPs’ experiences, prioritization and perspectives on treatment and follow-up of patients with cancer and comorbidity.

## Methods

### Study design

A qualitative study based on semi-structured interviews with GPs in Denmark. This study is part of a larger study which includes interviews with patients, and a description of the study design and method has also been reported earlier [25]. Cancer was the index disease, and DM, CVD and COPD were the inclusion comorbidities.

### Setting

The study was carried out in a general practice setting in Region Zealand, Denmark. Almost the entire Danish population is registered with a GP, and treatment is tax-financed and free for patients [45]. The Danish College of General Practitioners develops clinical guidelines for chronic care with recommendations regarding the frequency and the content of these consultations [1]. The guidelines are formulated by working groups consisting of general practitioners and medical specialists from relevant specialties. In 2018, a new guideline on follow-up on certain types of cancer was disseminated [2]. According to this guideline, a structure of keeping in contact with the patient is highly recommended, as well as stringent attention to the management of comorbidities. Most patients with chronic conditions, such as DM, CVD, and COPD, receive treatment and follow-up in general practice according to disease-specific guidelines. For example, the guideline for COPD recommends that the severity and risk factors are assessed, and that treatment is planned based on the patient’s resources, preferences, and treatment goals. It is also recommended that the patient is followed - typically with quarterly check-ups but with a frequency depending on the severity of the condition. Extra consultations are, for example, offered if the condition has worsened or medication is changed. In addition, the patient can be referred to rehabilitation programs in the municipality. At least yearly, there must be a comprehensive consultation with an extended evaluation and examination program. The individual practices must describe their procedures for how the chronic care is operationalized in their practice, which also depends on which staff the practice has. Therefore, the exact structure of chronic care can, to some degree, differ between practices, including how systematic the practice is regarding reminding patients about their chronic care consultations. In many general practices, however, nurses and GPs work in “chronic care teams,” where the nurses perform the quarterly checks with back-up from the GP. At the time of this study, the comprehensive annual consultation had to be performed by the GP. Many practices have a rule that the annual consultation is in the patient’s birthday month, and to avoid loss to follow-up, ideally, patients do not leave a chronic care consultation without a new appointment for the next check-up. The chronic care consultations typically involve a discussion of lifestyle, control of biomedical measures, and initiation and adjustment of medical treatment. Further, the collective agreement between GPs and the Danish regions secured an extra fee for the extended chronic care consultation once a year [45].

### Data collection

#### Participants

The first author (ABA) contacted 51 general practices (97 GPs) in Region Zealand, who had previously been invited to participate in the education of medical students, and informed them by telephone about the project. ABA sent a written description of the study to the 35 practices (76 GPs) that showed an interest in participation. To participate in the study, GPs had to recruit at least one patient with non-metastatic cancer, who had completed primary cancer treatment within the last five years, and who had one of the inclusion comorbidities. Two GPs included two patients each. We strategically selected GPs who expressed an interest in taking part, aiming for variation in gender, age, seniority in practice, practice type (single-handed/partnership), and location [46]. Inclusion of GPs ended when we reached data saturation as regards both the GPs and their included patients. Thirteen GPs from nine practices participated in the study (Table 1). The reasons the GPs gave not to participate were busyness.

**Table 1:** GP profiles

Age, years, mean (range)	56	(43–70)
Gender, male/female (n)	7/6	(13)
Seniority, years, median (range)	19.5	(7–34)
Practice type		
1 GP	1	
2–3 GPs	11	
> 3 GPs	1	
Practice location, inhabitants		
**<** 5000 (rural)	4	
5.000–20.000 (semi-urban)	1	
> 20.000 (urban)	4	

Characteristics of participating GPs

#### Interviews

In 2016 ABA, a GP trainee and ph.d. fellow, conducted single individual, semi-structured interviews with each of the selected GPs (*n* = 13). The interviews took place in the GP’s clinic and only the GP and ABA were present during the interviews. The interviews lasted 25–50 min, and an interview guide provided a flexible framework for questioning the GPs’ role and their experiences of and perspectives on chronic care consultations with patients before, during and after the cancer diagnosis. The interviews’ starting point was the patient(s) the GP had included in the study. During the interview process, the interviews could be extended for the GPs to include their experiences regarding other similar patients. All interviews were audio-recorded and field notes were made immediately after the interviews.

#### Data analysis

**Fig. 1:**
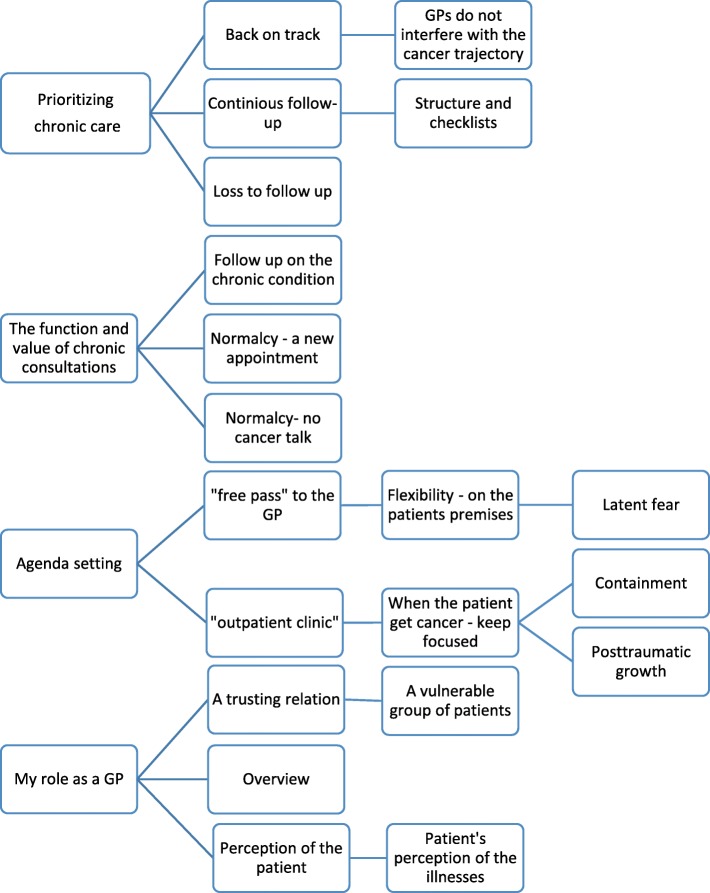
Coding tree

All interviews were transcribed verbatim. Data we analyzed using Systematic Text Condensation [47], which is inspired by phenomenological thinking and represents a pragmatic approach. The procedure consists of four steps: 1) Total impression of the whole dataset. Reading through all material and noting preliminary themes with an open mind. 2) De-contextualization. A flexible process where we identified, sorted and coded text fragments (meaning units) into code groups which contained information about our research questions. 3) Condensation. We sorted the meaning units into subgroups and reduced the content into coherent text amalgamations. 4) Synthesizing. We used coherent condensates and quotations from the subgroups and code groups to develop an analytical text grounded in the empirical data. A coding-tree can be seen in Fig. 1.

The method is inductive and not theory-driven with predetermined categories. Nvivo11 was used to facilitate coding and analysis. The research team included medical doctors with GP training. Initial coding of data was conducted by ABA after discussing the identification of themes, especially with ASD, who also read all the data material. After that, ABA performed the stepwise analysis in cooperation with ASD, and every step was validated against the full data material, by re-reading and re-coding several times. The findings were finally reflected upon and discussed among all the researchers until we reached consensus.

#### Ethical considerations

Written consent was obtained from all participants before the interviews. The Regional Committee for Health Research Ethics in the Capital Region assessed the study and stated that, according to Danish legislation, this study needed no approval (journal no. H-15019197). The study was notified to the Danish Data Protection Agency.

## Results

The GPs gave chronic care consultations a high priority independent of the patients’ history of cancer. If cancer treatment interrupted the care of the patients’ comorbidities, the GPs said that the consultations regarding chronic care were soon put “back on track.” They experienced that only very few patients were lost to follow-up.

The GPs described two different types of approach to chronic care consultations. Some said that they followed an agenda with a focused structure based on clinical guidelines for the chronic condition; whereas others described a more flexible approach, which was more attentive to the patient’s agenda. These GPs experienced that chronic care consultations could also serve as an opportunity for patients to talk about more sensitive issues, e.g. issues related to their cancer.

### Prioritizing chronic care in patients with a history of cancer

#### Continuous care

Chronic care consultations were highly prioritized by the majority of the GPs independent of the patient’s cancer diagnosis. They said that because the cancer treatment and control were carried out in hospitals, and chronic care in general practice, treatments were performed in parallel. Regarding control of chronic conditions, the GPs described a very systematic and well-organized structure in general practice, which ensured that patients remained in the chronic care process. GPs, nurses and other staff in general practice were responsible for adhering to this structure, and the practice secretary would contact patients who did not attend the planned chronic care consultation.

*“The diabetes consultations are so well-integrated, so it takes a lot for it to fail … otherwise, they [the patients] are called in, regardless of whether they also have cancer or not. Those patients where we can say, ‘this is not good enough’ – those we call in.”* (GP 2)

Having a structure for chronic care consultations was highly valued by the GPs and considered necessary for keeping up contact with the patients. This was especially true for patients who had been absent for a time due to cancer treatment. The GPs viewed continuous chronic care appointments as a way of reducing the risk of losing contact. Some of the GPs experienced that the hospitals did not attend to the treatment of the patient’s chronic conditions, therefore it was important to avoid losing contact with these patients.

#### Back on track

The GPs reported that the frequency of chronic care consultations could decrease during cancer treatment. Chronic care receded into the background for a while and, in accordance with the patients’ preferences, the focus on chronic conditions could temporarily be given a lower priority.

*“It’s cancer that sets the pace for how long we go with the chronic illnesses and how aggressive we are with treating them [the patients].”* (GP1)

However, the GPs described that when the intensity of the cancer treatment decreased, and the patient recovered both physically and mentally, attention to the comorbidities would come back on track, both regarding the frequency of consultations and the attention to lifestyle. If the patients did not return for chronic care, practice staff would resume the contact. GPs experienced that resuming chronic care consultations was easier if these consultations had been regular before the patient was diagnosed with cancer.

*“Everything else will be given a lower priority but then it is as if it comes back to a normal level, and then we find our level again.”* (GP 13)

*“Actually I think, that, regarding treatment goals or thoroughness, we treat patients with chronic conditions very carefully irrespective of if they have a malignant disease or not.”* (GP 4)

Many GPs said that even when the frequency of contacts had to be adjusted to accommodate cancer treatment for some time, it was important to continue the chronic care appointments to maintain a treatment alliance with the patients. Furthermore, the GPs experienced that their prioritization of and engagement in chronic care had a positive impact on the patient’s engagement. GPs felt that most patients appreciated the chronic care consultation because they saw that their health was taken seriously.

### Who sets the agenda?

The GPs described their approach to carrying out chronic care consultations in two different ways. Some said that they perceived the chronic care consultations as imitating consultations in a hospital outpatient clinic. In this approach, the structure and content of the consultations were defined by the GP’s agenda. Other GPs described a more flexible approach and said that they were also open to the patient’s agenda.

#### Chronic care consultations imitating outpatient clinics

In this type of consultation, the GP set the agenda to keep focus. Patients could be prepared for the content of the chronic care consultation using both spoken and written information. The consultation was performed in a very structured way according to clinical guidelines, templates and specific phrases in the patient’s medical record. For the GPs who followed this approach, it was important that chronic care consultations were effective and proceeded according to the predetermined agenda.

*“I am mostly very systematic – because I have a phrase in my record system which tells me that I have to go through such and such items, and then I say to the patient just when they arrive, we have to go through a checklist of items. I just go through it, and we have to manage”* (GP13)

According to these GPs, talking about cancer was not considered a relevant topic as it was not the purpose of the chronic care consultation. If the patient brought up cancer-related issues, these were politely postponed to another consultation, which sent a clear signal about the importance and prioritization of chronic care.

*“Actually, I think that it’s colliding a bit, if you sit and perform an important annual chronic consultation, which is already crammed with all sorts of items, and you then have to go through whether you should also talk about complications or fear of cancer… I would not spend my time on that… so, I say to the patient, that this appointment corresponds to the outpatient clinics at the hospital in the old days, which now I have taken over… so, I will need to keep focused and ensure that everything is under control”* (GP 6)

#### Chronic care consultations as an “alibi”

The GPs who described a more flexible approach to chronic care consultations said that their patients did not distinguish between a chronic care consultation and a regular appointment, and therefore they would bring up whatever was on their minds.

“*The patients do not look at it like they show up to talk about COPD – they are just going to the GP… well, and I think that we are going to have an annual chronic care consultation.. but then all the other questions arise…We are not so rigid; we talk about all sorts of stuff…Sometimes I just let them [the patients] set the agenda for what is important – instead of running with my agenda.”* (GP5)

Some GPs described that some patients used chronic care consultations as an “alibi” to talk about more difficult and sensitive subjects, e.g., anxiety regarding cancer or other subjects of concern. The GPs explained that talking about difficult issues could be perceived by patients as easier in a consultation that was scheduled in advance, than if the patients had to take the initiative and consult the GP about something that was troubling them.

*“The chronic condition they have gives them like a ‘free pass’ to come up and talk with me … actually, those of my patients who have cancer, they use just as much of their time in the chronic care consultation to talk about cancer, and it is difficult for me to tell them not to. And I myself cannot help not asking about cancer – it is natural – and I cannot just be so rigid… Actually, if I have a patient with both chronic conditions and cancer, then I should be extremely hard-pressed to ignore it [cancer] – it is always on the agenda.”* (GP1)

Some GPs used chronic care consultations as an opportunity to ask about the patient’s cancer process and to supplement discharge summaries from hospitals if the patient was still in the follow-up phase. One doctor narrated that one of her patients explicitly asked the GP to always mention cancer in the consultations to ensure that it was taken into account. In some patients, the former cancer illness took the form of “dormant fear,” and some GPs explained that, for these patients, the absence of cancer-talk could induce fear. Therefore, to acknowledge that the patient had been through a tough period, perhaps with physical and mental changes, some GPs made sure to ask about cancer as a natural part of the chronic care consultation.

### The value of chronic care consultations

#### Normalcy and positive expectations

According to the GPs, most of the patients attended the practice regularly for chronic care consultations. It was the GPs’ impression, therefore, that these appointments represented a symbol of “normalcy” and “everyday life” – particularly in the light of the previous exhausting cancer process. However, the GPs’ arguments for maintaining this sense of normalcy differed. Some were very conscious about planning future chronic care consultations because both the quarterly and the annual contacts, and the mere appearance of the patients in the practice, were perceived as an anchor and a symbol of a well-known, everyday routine. Further, by making a new appointment for the next chronic care consultation, there was an embedded positive expectation for the patient’s prognosis.

“*I have learned from experience. If I just send the patients out the door, well that is actually the same as ‘sending them to their death’, metaphorically speaking…so, they [the patients] like that there is some normalcy, so, I say, ‘of course you will have a new appointment.’”* (GP 1)

Another way to support normalcy was to focus on the chronic condition and deal with the cancer as cured. Some GPs said that confronting patients with their former cancer experience was not needed, because patients might want to distance themselves from the role of being a “cancer patient.” This group of GPs, therefore supported normalcy by not talking about cancer to signal that cancer was a past phenomenon and that the patient was now treated the same as any other patient.

*“…Well, it is not like we angle for a ‘cancer-agenda’ when we perform an annual diabetes consultation… I actually think, that at some point in time you need to say: ‘well, I live with having had cancer’… I think that a lot of people … don’t want to think of it in their daily life…”* (GP 6)

## Discussion

### Summary of main findings

All the participating GPs said that they gave a high priority to chronic care in patients with a history of cancer. Two different approaches to chronic care consultations were identified: some GPs described this kind of consultations as imitating hospital outpatient clinics, with the GP setting the agenda. Other GPs described an approach that was more attuned to the patient’s agenda.

GPs experienced that chronic care consultations supported normalcy in different ways. Some GPs supported normalcy by giving the patients future appointments on the same terms as patients without cancer. Other GPs strove for normalcy by focusing exclusively on the chronic condition and dealing with cancer as cured.

#### Comparison with existing literature

All GPs made a substantial effort to keep in contact with their patients through structured practice organization, and they experienced that only very few patients were lost to follow-up. Other researchers, however, have described that discontinuity in chronic care is frequent when patients have cancer and multiple chronic diseases [12]. This discontinuity can be self-perpetuating if patients experience difficulties in resuming contact with their GP [12, 13], which could result in lower use of health care [15–18]. The present study, however, showed that all participating GPs gave a high priority to chronic care for all patients, which seems to fulfill the patients’ needs because they experienced chronic care consultations as important, regardless of their previous cancer diagnosis [25]. It seems that considerable attention was paid to chronic care which might be due to an above-average involvement by the GPs included in the study. The attention to chronic care might, however, also be influenced by the economic incentive to GPs [45].

Some GPs were more likely to follow the patient’s agenda and allow talk about other issues, e.g., cancer; while other GPs set an agenda with a focused structure based on the clinical guidelines. Clinical guidelines might, therefore, have effects on the content of the consultations.

Lippert et al. [48] argue that strict adherence to standards and clinical guidelines can narrow the focus of doctor-patient dialogue and lead to a more quantitative and biomedical view, away from “real” patients with “real” concerns. This might hinder patients from bringing up other issues of concern because they will be turned into members of a disease category instead of unique patients with a story to tell [36, 41, 49, 50]. GPs may thus be blinded by a narrowed biomedical focus [51] which carries a risk of patients leaving the consultations with unmet needs [52].

The focused agenda that mirrors an outpatient clinic is in contrast to the other approach, which is more attuned to the patient’s agenda and reflects the old “brand” of GPs as having a holistic, patient-centered and biopsychosocial approach [29, 53–55]. In these cases, GPs value general practice as “being different” from the secondary sector [28] in the sense of aiming to avoid fragmented care. However, the two approaches represent the extremities on a spectrum, and most GPs might approach chronic care consultations somewhere within this spectrum.

#### Normalcy and chronic care consultations

For patients who had been living with chronic conditions for many years before being diagnosed with cancer, chronic care consultations had become a symbol of normalcy and part of the everyday routine [25]. Visiting the GP for a chronic care consultation could be considered as a form of *ritual* by patients. The concept of rituals was coined by Goffman and might elucidate the symbol of routines from a theoretical perspective. Goffman describes the seemingly mundane features of everyday life - the ordinary*,* including rituals and routines *-* such as a hand-shake greeting or a wave good-bye [56]. This is in line with how patients describe their appreciation of the everyday nature of their contact with general practice and the relationship they have with their GP and the practice staff [25]. Goffman was especially interested in *ratifactory rituals*, which are used when people have changed physically or mentally, to show that “everything is by the old” e.g. as regards the relationship. Therefore, when the GPs make the next appointment in practice, this can be seen as a symbol of normalcy and a ratifactory ritual because, despite possible physical and mental changes, everything is “by the old” regarding the patient’s relationship with their GP and the practice.

#### “Cancer-survivor”

The patients in the present study had completed primary cancer treatment and could be considered “cancer survivors.” However, [57] patients interpret the term “cancer survivor” different. In the present study, GPs said that some patients asked for their cancer disease to be mentioned at chronic care consultations, to make sure that the GP took it into account. On the other hand, other patients did not want to be labeled as cancer patients. This finding is in line with other studies, which show that some patients identify with the “survivor identity,” as the cancer experience has been an important part of their lives, whereas others reject this identification and want to move on and avoid unnecessary focus on the disease [25, 57–59]. Whether or not cancer or other matters of concern to patients were taken into account during chronic care consultations in the present study depended on the GP’s prioritization.

Employing a patient-centered approach demands that the GPs have knowledge of the patient’s life and further, the patient’s ability and opportunity to disclose their own agenda. As more tasks are being outsourced from hospitals to general practice, including chronic care, some GPs in the present study talked about the experience of being forced into following a focused structure in the chronic care consultations. Therefore, the agenda might not be set by the patient nor the GP but by the guidelines. This could, therefore, carry a risk that care in general practice will become more fragmented too. This possible fragmentation and single-disease focus might not only collide with patients’ expectations, but also with GPs’ conception of what it is to be a GP, with a possible ongoing impact on GPs’ job satisfaction [60].

Therefore, it is important for GPs to hold on to their professional identity and strive to maintain a patient-centered approach that focuses on the whole person instead of a single disease. This study shows that patients might use chronic care consultations to disclose other issues of concern. It could, therefore, be questioned whether chronic care consultations for comorbidities are sufficient to meet the needs of patients with a history of cancer. The new guideline for follow-up on cancer in general practice [61] emphasizes the importance of consultations with the GP and of obtaining the patients’ narrative and perspectives. This means that if patients’ concerns about the cancer cannot be discussed in the chronic care consultation, the GP should be aware of offering a separate consultation to fulfill these needs.

### Strengths and limitations

The characteristics of the GPs who participated in the study varied widely, which is a strength meaning that our findings could apply to GPs in other Danish regions. Another strength is that several authors participated in the analysis of data and discussed results until we reached consensus. As all authors are GPs/GP trainee, professional loyalty might have hindered deeper elaboration of certain issues, as knowledge can be taken for granted due to shared medical and professional understanding. The researcher’s pre-conceptions, knowledge from literature and experience from everyday clinical work will unavoidably influence the analysis and interpretations. If we had included interviews with nurses in general practice, they may have contributed with other responses to our subject matter.

On the other hand, GPs could align themselves with the interviewer as a peer and a professional colleague, and thus they may have been more responsive and encouraged to disclose different issues [62]. Interviewing peers may influence the content of data in different ways, e.g., GPs could view the interview as a test of their knowledge or scrutiny of their practice, although we reassured them to the contrary. This could result in the possibility that GPs provided more idealized answers [63] regarding the organization of chronic care in their practices.

GPs in our study may have an above-average involvement in the care of people with cancer and comorbidity, which one should bear in mind regarding the GPs’ opinion that patients lost to follow-up was rare. Nevertheless, the GPs in the sample represented different perspectives on how chronic care consultations should be carried out, and as the GPs used a patient case as a starting point, the interviews were clinically anchored. We think, therefore, that the results are valid and could be transferred to other GPs in Denmark and in countries with similar primary care systems.

## Conclusion

All participating GPs reported that they gave high priority to chronic care in patients with a history of cancer, and they emphasized the importance of continuous chronic care appointments to maintain contact with the patients and to inspire hope and positive expectations to the patients’ prognosis.

Two different approaches to chronic care consultations were described: one group of GPs prioritized the planned and structured agenda regarding the chronic condition. The other group described an approach which was more attuned to the patient’s agenda and often involved aspects related to the cancer diagnosis – in these cases, chronic care consultations could serve as an alibi to bring up other issues of concern.

GPs said that chronic care consultations supported normalcy in different ways. One way was to be conscious about planning future chronic care consultations on equal terms as for patients without cancer. Another way to support normalcy was to focus exclusively on the chronic condition and to consider that the cancer was cured. A strong focus on a structured single disease approach might, however, collide with the core values and patient-centeredness of general practice, and this could place some GPs in a dilemma regarding their professional identity and job satisfaction.

### Practice implications and future research

According to the GPs in the study a clear structure regarding chronic care follow-up is fruitful in keeping up contact with patients with cancer. Therefore, to decrease the risk of discontinuity, GPs and practice staff should continue to be proactive in offering and planning future consultations. Further, GPs should emphasize to their patients that the GP is available to them during the cancer treatment process, and in the period after completion of treatment.

Moreover, patients will not necessarily attend chronic care consultations with only one issue, and their agenda might differ from the GP’s planned agenda. Chronic care consultations, therefore, might serve other purposes than just biomedical control. GPs should be aware that a strict structure, which primarily focuses on biomedical parameters, might present a barrier to establishing a relationship with patients that goes beyond the facade, and this might prevent patients from disclosing additional problems or issues that are troubling them, including issues related to their cancer diagnosis and treatment.

As clinical guidelines typically refer to single diseases, treating patients with multiple diseases might result in an increased risk of fragmented care and hamper a whole-person approach. Turning general practice into an “outpatient clinic” by focusing on single diseases in this way might collide with the core values of general practice, the GPs’ self-perception and job satisfaction, and might reduce the quality of care provided in general practice. GPs should be aware of possible conflicts and tensions regarding the content, and patients’ expectations, of chronic care consultations in general practice.

To analyze the in situ interaction in the chronic care consultations, and how the GP and the patient prioritize and negotiate the content and the agenda of the consultation, further observational research is required. Video-recordings of consultations could contribute to studying the interaction between doctors and patients.

## Data Availability

The datasets used and analyzed during the current study are available from the corresponding author on reasonable request.

## Abbreviations

COPD: Chronic obstructive pulmonary disease
CVD: Cardiovascular disease
DM: Diabetes mellitus
GP: General practitioner
